# A COVID-19 case report from asymptomatic contact: implication for contact isolation and incubation management

**DOI:** 10.1186/s40249-020-00686-3

**Published:** 2020-06-19

**Authors:** Chang-Qiang Zhu, Shi-Ding Gao, Yan Xu, Xiao-Hong Yang, Fu-Qiang Ye, Le-Le Ai, Rui-Chen Lv, Bin Zhang, Yin Li, Heng Lv, Yun-Sheng Liu, Chun-Meng Shi, Chun-Hui Wang, Wei-Long Tan

**Affiliations:** 1 Centre for Diseases Prevention and Control of Eastern Theater, Nanjing, 210002 China; 2 The No: 908 Hospital, Nanchang, 330000 China; 3 Centre for Diseases Prevention and Control of Jiangsu Province, Nanjing, 210009 China; 4 Army Medical University, Chongqing, 431000 China

**Keywords:** COVID-19, Coronavirus, Infection, Particular contacting

## Abstract

**Background:**

As of 2 March, 2020, at least 80 151 coronavirus disease 2019 (COVID-19) cases were reported in China. Most of the patients had a history of visiting Hubei Province or contacting with people who had ever stayed in or passed by Hubei Province or were exposed to symptoms. Some patients got infected through only asymptomatic contact. This study aimed to report the epidemic features and lab identification of a patient confirmed with COVID-19 infection through only asymptomatic contact.

**Case presentation:**

A 44-year-old man, who lived in Nanchang, Jiangxi Province, China until 6 March 2020, suffered from cough on 27 January 2020. Fever symptoms appeared on 28 January, with a maximum temperature of 38.8 °C, accompanied by cough, sore throat, headache, fatigue, muscle ache, joint ache, and other symptoms. The symptoms continued until he was hospitalized on 30 January. Coronavirus conventional polymerase chain reaction assay was positive for the throat swab sample. The patient, along with his wife and son, drove from Nanchang to back to Honghu City, Hubei Province, on 23 January 2020. After staying with his parents and brother’s family for 3 days, the patient drove back to Nanchang and arrived on 25 January. On the way back home, they stopped by Tongshan service area, Hubei Province, without any close contact with other people. After arriving home in Nanchang City, Jiangxi Province, none of them left their residence. In addition, his parents stayed at home for 20 days with his younger brother’s family before they got back. His younger brother and one of his brother’s children visited Wuhan on 5 January and came home on 6 January 2020.

**Conclusions:**

This report suggested that, in the early phase of COVID-19 pneumonia, routine screening could miss patients who were virus carriers. Highlighting travel history is of paramount importance for the early detection and isolation of severe acute respiratory syndrome coronavirus 2 cases.

## Background

**Fig. 1:**
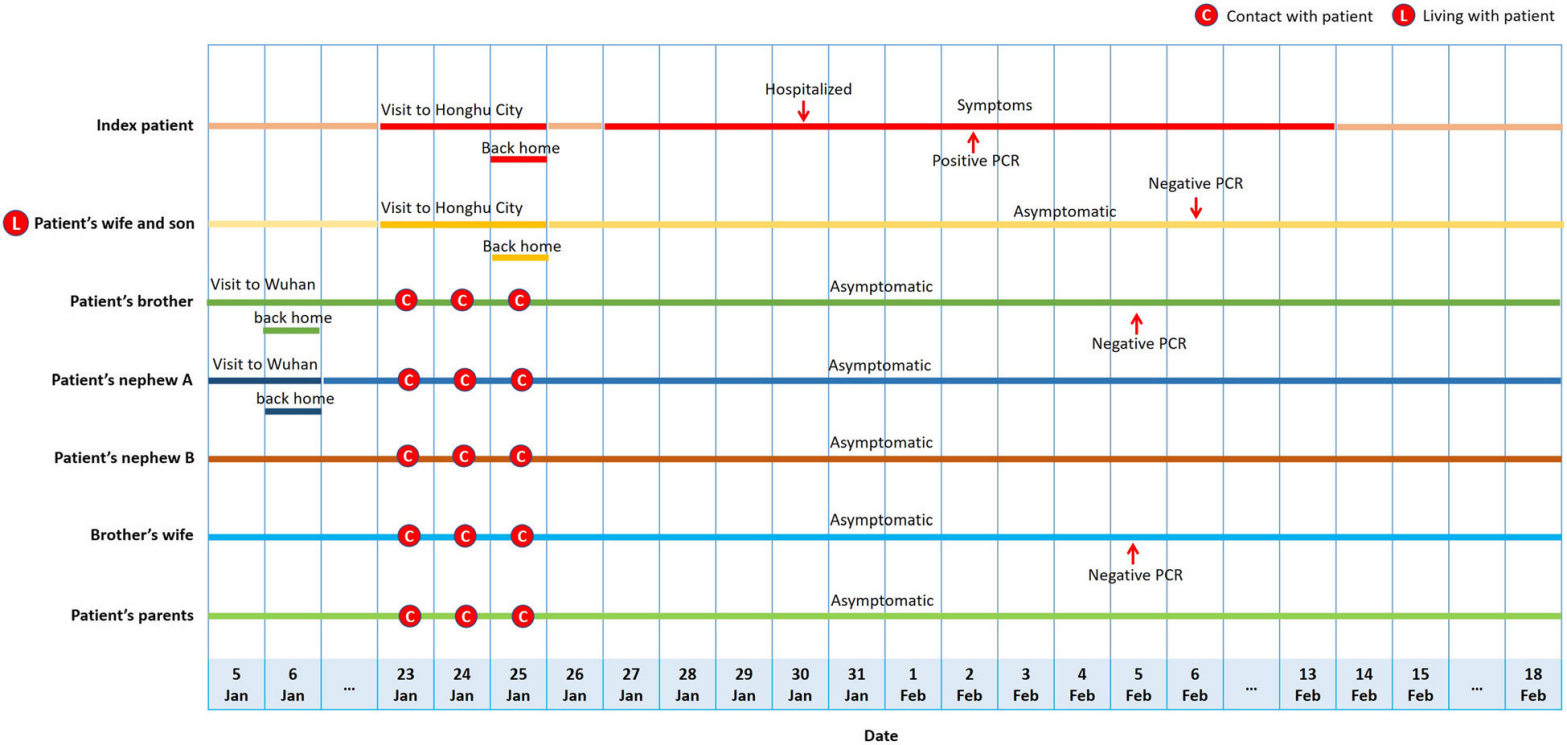
The detailed onset timeline of the index patient

Coronavirus (CoV) is a positive-sense single-stranded RNA virus [1]. Many kinds of mammals, such as hedgehog, pangolin, civet, and bat, can serve as storage hosts of coronavirus [2–6]. Six CoVs have been identified to be pathogenic, including four endemic (HCoV-OC43, −229E, −NL63, and -HKU1) and two epidemic (SARS-CoV and MERS-CoV) viruses [7, 8]. A cluster of cases of pneumonia was reported in Wuhan, Hubei Province, China, in December 2019. On 11 February 2020, the diseases named as coronavirus disease 2019 (COVID-19) by the World Health Organization (WHO), and Coronavirus Study Group (CSG) of the International Committee proposed to name the new coronavirus as SARS-CoV-2. Up to 2 March 2020, at least 80 151 cases were reported (http://www.gov.cn/xinwen/2020-03/03/content_5486171.htm). Most of the patients had a history of visiting Hubei Province or contacting people who had ever stayed in or passed by Hubei Province, or were exposed to symptoms [9]. Some patients got infected through only asymptomatic contact. This study aimed to report the epidemic features and lab identification of a patient confirmed with COVID-19 infection through only asymptomatic contact (Fig. 1).

## Case presentation

**Fig. 2:**
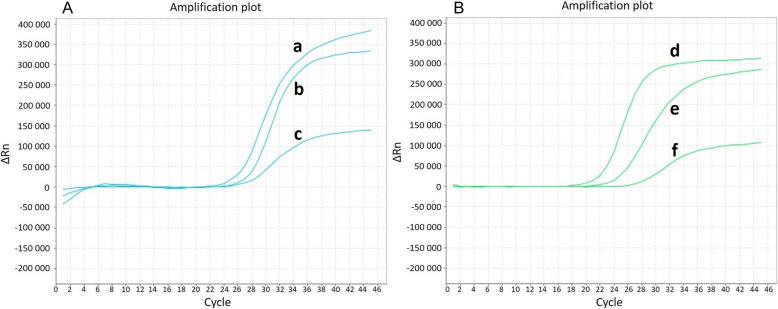
Imaging of the patient using reverse transcription PCR. **A** Positive control of ORF1ab (a), N (b), and E (c). **B** Specimen detection of ORF1ab (d), N (e), and E (f)

A 44-year-old man, who lived in Nanchang, Jiangxi Province, China, got a fever of 38.8 °C on 27 January 2020. He wore a mask and drove himself to see a doctor in a local hospital in Nanchang on 30 January 2020. He was immediately hospitalized in an isolation room. The PCR assay result was positive for the throat swab sample using a SARS-COV-2 real-time RT-PCR Kit (Fig. 2). The patient was an instructor in a university and visited the city of Huangmei, Hubei Province. He stayed with his parents and his brother’s family, but denied any exposure to a febrile patient or wild animals, or visits to wet markets, including the Seafood Market in Wuhan.

Before the onset of symptoms, the patient, along with his wife and son, drove from Nanchang to Honghu City, Hubei Province, on 23 January 2020, where his parents and brother lived. They did not stop by any of the expressway service area. After arriving, they stayed at home and did not visit any public place in Honghu City. In addition, his parents stayed at home for 20 days with his younger brother’s family before they got back. His younger brother and one of his brother’s children visited Wuhan on 5 January and came home on 6 January 2020. After staying with his parents and brother’s family for 3 days, the patient drove back to Nanchang and arrived on 25 January. On the way back home, they stopped by Tongshan service area, Hubei Province, without any close contact with other people. After arriving home in Nanchang City, Jiangxi Province, none of them left their residence.

**Fig. 3:**
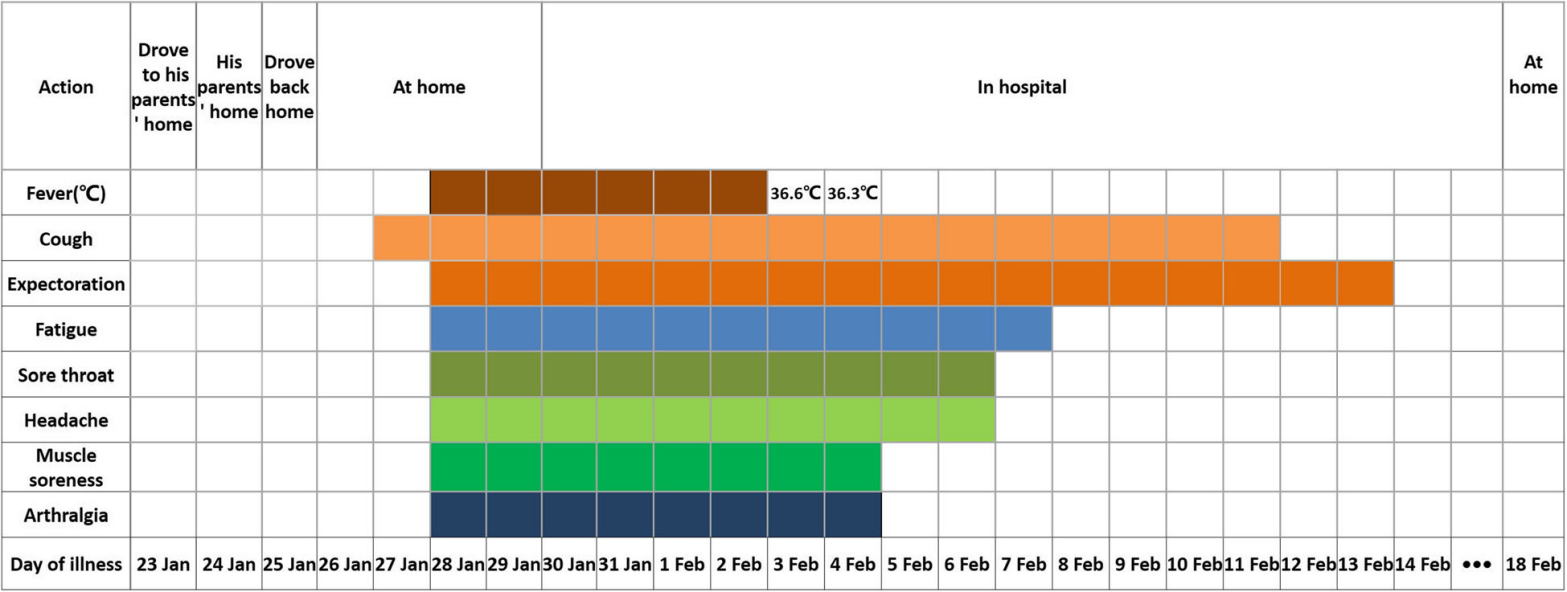
Symptoms and maximum body temperatures according to the day of illness between 27 January and 18 February 2020

**Fig. 4:**
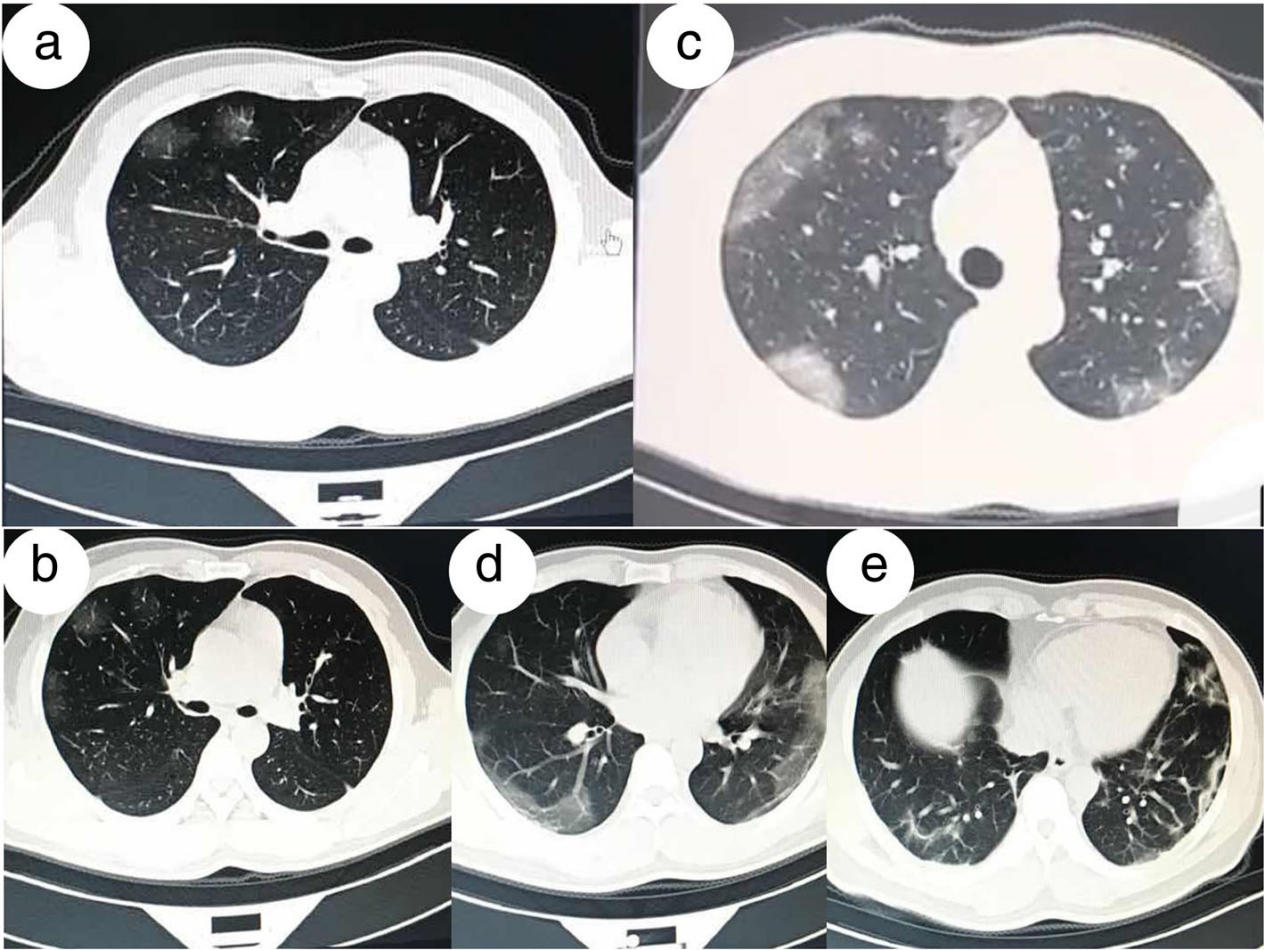
Chest imaging of the patient. **a** and **b** Chest CT scans of the lung on 30 January and 31 January 2020 (3–4 days after the symptom onset) revealed slight pulmonary infiltration. **c** and **d** CT scans taken on 3 February 2020 (8 days after the symptom onset) showed obvious pulmonary infiltrates. **e** CT scans taken on 10 February 2020 (15 days after the symptom onset). HRCT, High-resolution computed tomography

The patient began to have a cough on 27 January 2020, and took cefalexin capsules by himself. Fever symptoms appeared on 28 January, with a maximum temperature of 38.8 °C, accompanied by cough, sore throat, headache, fatigue, muscle ache, joint ache, and other symptoms (Fig. 3). The symptoms continued until he went to the doctor. At the hospital’s fever clinic, he underwent blood routine examination and lung computed tomography (CT) examination (Fig. 4).

He was not obese and had no previous basic disease. On admission (30 January 2020), the physical examination revealed a body temperature of 38.8 °C, a respiratory rate of 19–21 breaths per minute, a pulse of 62–88 per minute, and a blood pressure of 76/128 mmHg. The initial chest radiography showed a glass density shadow of both lungs (Fig. 4a-b).

On 31 January 2020, the blood routine examination revealed the following: white blood cell (WBC) count: 7.4 × 10^9^/L; lymphocyte (LY) count: 0.6 × 10^6^/L. Another routine test on 31 January showed the WBC count of 4.4 × 10^9^/L and a decreased C-reactive protein level of 4.9 mg/L. The lung CT examination at 8:00 PM showed lung lesions significantly larger compared with those on the previous day.

However, high-resolution computed tomography on 3 February 2020 (day 8 of illness), revealed multiple, ground-glass opacities located in both subpleural spaces (Fig. 4c-d). The blood routine examination showed mild changes in neutrophil and lymphocyte counts. During admission, he developed nasal congestion, cough, and pleuritic chest discomfort.

He received the following medicines treatment during hospitalization for 18 days. Including: oral lopinavir/ritonavir (6.67/1.67 mg/kg/day [12 h]), aerosol inhalation of interferon alpha (0.833 μg/kg/day [24 h]), oral lianhuaqingwen capsule (23.33 mg/kg/day [8 h]), oral suhuang zhike capsule (22.5 mg/kg/day [8 h]), enteral nutrition (0.833 g/kg/day [6 h]), thymalfasin for injection (26.67 μg/kg/3 days [72 h]), xuebijing injection (1.67 ml/kg/day [12 h]). In addition, the hormone was only used for 5 days, methylprednisolone sodium succinate (0.667 mg/kg/day [24 h]) for 3 days, methylprednisolone sodium succinate (0.333 mg/kg/day [24 h]) for 1 day, methylprednisolone sodium succinate (0.167 mg/ kg/day [24 h]) for 1 day, and then stopping use hormone.

## Further tracing of the source of virus

**Fig. 5:**
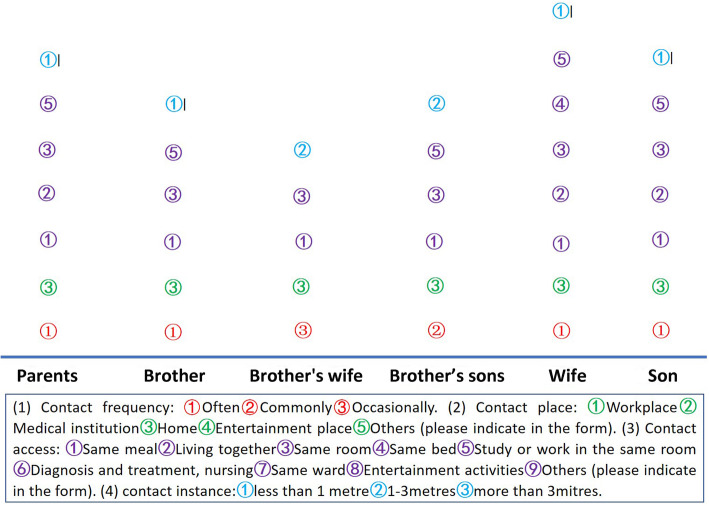
Contact styles of the patient with his relatives and families

**Fig. 6:**
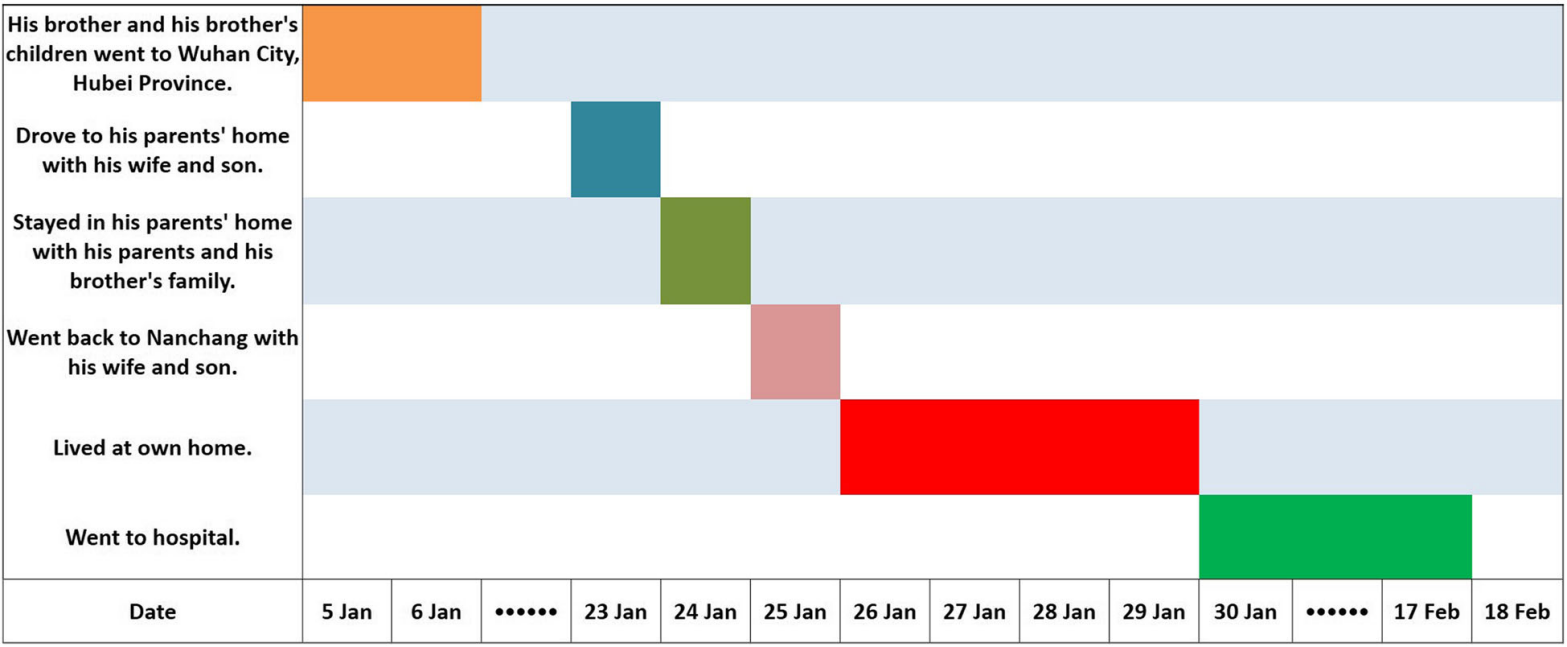
Travel and residence history of the patient and his family

Before the patient’s visit to Honghu, his relatives, including his parents and brother’s family, had no disease or infection through contact or clustering. After he was hospitalized, the results of the coronavirus conventional PCR assay of his wife, son, brother, and brother’s wife performed once every other day were negative. No farmer’s market was located near their residence. The patient did not go to any farmer’s market. His wife and son, who were two close contacts with a final contact on 30 January 2020, had no symptoms or signs till 18 February 2020 (Figs. 5 and 6).

## Discussion and conclusions

This patient was diagnosed with SARS-CoV-2 infection. His PCR test was positive, but the test for seasonal influenza and adenovirus infection was negative, despite flu-like symptoms and pneumonia during the first week of illness. In this case report, the COVID-19 infection was diagnosed by a PCR test or based on clinical symptoms combined with a CT scan [10].

**Fig. 7:**
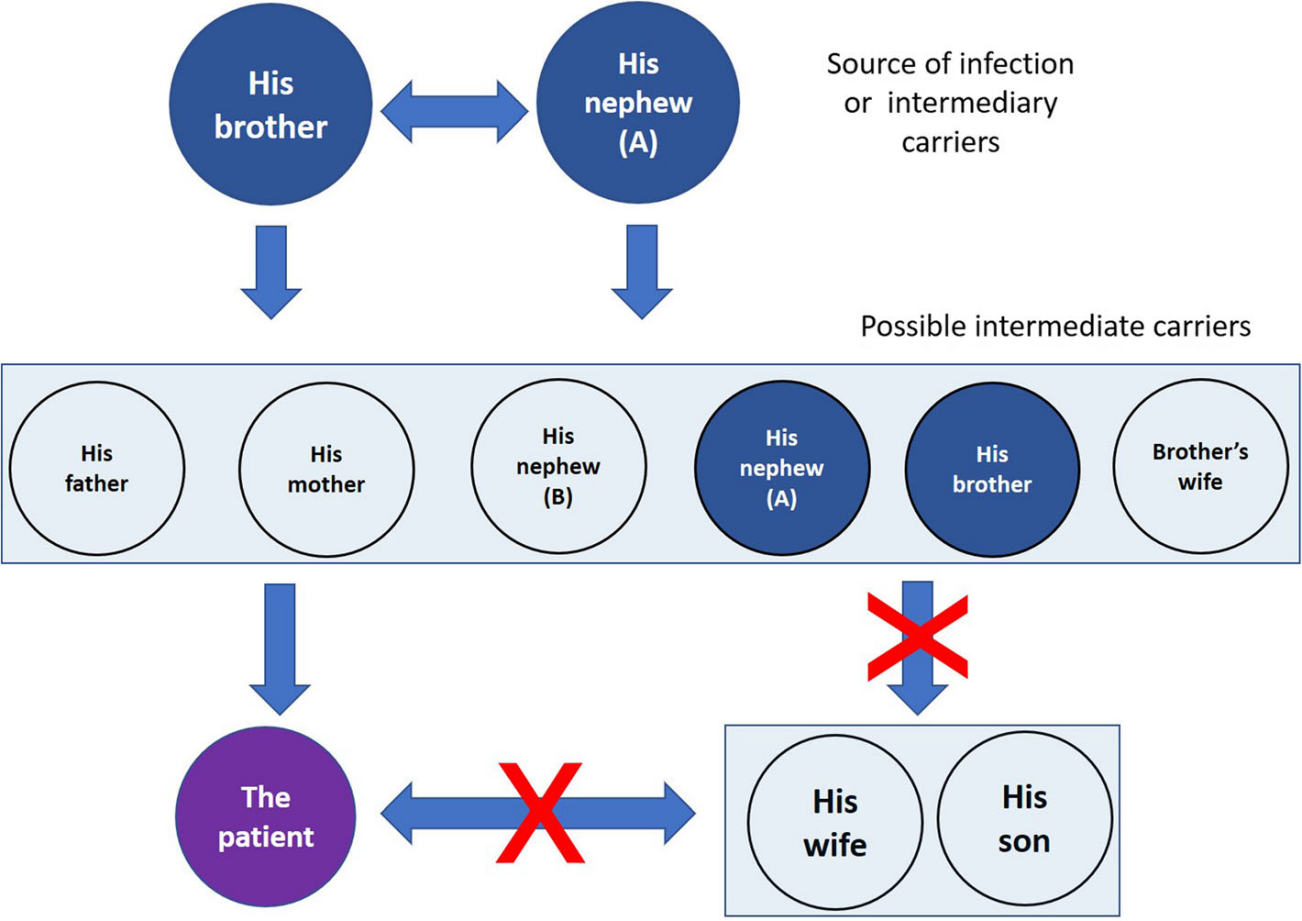
Possible transmission of the virus among relative

The duration from infection to the appearance of symptoms of SARS-CoV-2 was not exactly known [11]. Generally, it was thought to be 14 days, but a research group at Guangzhou reported the longest incubation period of 24 days [12]. In addition, asymptomatic transmission of SARS-CoV-2 is an important topic and asymptomatic infections of SARS-CoV-2 could complicate disease control [13]. The report of “Diamond Princess” showed that a large number of asymptomatic carriers might remain undiscovered in the community [14]. Mao et al. reported that the asymptomatic ratio of COVID-19 was 2.6% (2 out of 78 confirmed cases) [15]. Nishiura et al. estimated 41.6% of cases among 565 Japanese individuals evacuated from Wuhan, China were asymptomatic case [16]. As of 4 April 2020, 150 asymptomatic case out of 915 confirmed cases were reported in Hong Kong, giving a ratio of 16.4% [17]. Hu et al. provided evidence for transmission from an asymptomatic infector to close contacts that result in severe COVID-19 pneumonia [18]. These findings suggest that asymptomatic carriers should be considered a source of COVID-19 infection. In this study, the patient’s wife, son, parents, and brother’s family had no symptoms or signs between 5 January and 18 February 2020. After the patient was hospitalized, RT-PCR tests of his wife, son, brother, and brother’s wife performed once every other day were negative. Their PCR results were negative probably because the virus was eliminated due to the long-time interval or the viral load was too low to be detected, or the test results were false negative. Moreover, the patient’s parents and nephews A and B did not undergo the RT-PCR test for SARS-CoV-2 infection, and hence whether they were carriers was not confirmed. No suspected or confirmed cases were reported in Nanchang within 14 days of the departure of the patient from the site on 23 January. Therefore, it was difficult to predict who might have passed the virus to the patient; any of the relatives could have served as an intermediary of the virus except for his wife and son (Fig. 7).

This case report highlighted the possibility of developing SARS-CoV-2 infection through an asymptomatic contact. It indicated the need for contact isolation, especially for those who returned from the epidemic area without any symptoms [19, 20]. In addition, this report suggested that, in the early phase of COVID-19, routine screening could miss diagnosing patients who were virus carriers. The frequency of such transmissions of asymptomatic infection remains to be determined. The scale of transmission through an asymptomatic contact during the early phase of infection should be explored urgently.

In conclusion, this report indicated that highlighting travel history is of paramount importance for the early detection and isolation of SARS-CoV-2 cases. The findings might be used as a reference for the prevention and control during this global outbreak of COVID-19.

## Data Availability

All original data and materials are available from the corresponding author upon request.

## Abbreviations

CoV: Coronavirus
COVID-19: Coronavirus Disease 2019
CRP: C-reactive protein
CT: Computed tomography
HRCT: High-resolution computed tomography
LY: Lymphocyte
MERS: Middle East respiratory syndrome
nCoV: Novel coronavirus
PCR: Polymerase chain reaction
PM: Post meridiem
SARS: Severe Acute respiratory syndrome
WBC: White blood cell
